# Toxoplasmosis and Epilepsy — Systematic Review and Meta Analysis

**DOI:** 10.1371/journal.pntd.0003525

**Published:** 2015-02-19

**Authors:** Edgard B. Ngoungou, Devender Bhalla, Amandine Nzoghe, Marie-Laure Dardé, Pierre-Marie Preux

**Affiliations:** 1 INSERM, UMR1094, Neuroépidémiologie Tropicale, Limoges, France; 2 Université de Limoges, UMR 1094, Tropical Neuroepidemiology, Institute of Neuroepidemiology and Tropical Neurology, CNRS FR 3503 GEIST, Limoges, France; 3 Département d’Epidémiologie-Biostatistiques et Informatique Médicale (DEBIM/EA NEMIT), Faculté de Médecine, Université des Sciences de la Santé, Libreville, Gabon; 4 CHU Limoges, Laboratoire de Parasitologie-Mycologie, Limoges, France; 5 CHU Limoges, CEBIMER, Limoges, France; University of Chicago, UNITED STATES

## Abstract

**Background:**

Toxoplasmosis is an important, widespread, parasitic infection caused by *Toxoplasma gondii*. The chronic infection in immunocompetent patients, usually considered as asymptomatic, is now suspected to be a risk factor for various neurological disorders, including epilepsy. We aimed to conduct a systematic review and meta-analysis of the available literature to estimate the risk of epilepsy due to toxoplasmosis.

**Methods:**

A systematic literature search was conducted of several databases and journals to identify studies published in English or French, without date restriction, which looked at toxoplasmosis (as exposure) and epilepsy (as disease) and met certain other inclusion criteria. The search was based on keywords and suitable combinations in English and French. Fixed and random effects models were used to determine odds ratios, and statistical significance was set at 5.0%.

**Principal findings:**

Six studies were identified, with an estimated total of 2888 subjects, of whom 1280 had epilepsy (477 positive for toxoplasmosis) and 1608 did not (503 positive for toxoplasmosis). The common odds ratio (calculated) by random effects model was 2.25 (95% CI 1.27–3.9), p = 0.005.

**Conclusions:**

Despite the limited number of studies, and a lack of high-quality data, toxoplasmosis should continue to be regarded as an epilepsy risk factor. More and better studies are needed to determine the real impact of this parasite on the occurrence of epilepsy.

## Author Summary

Toxoplasmosis is a common parasitic infection and affects one-third of the global population. The burden this figure represents clearly signifies the public health relevance of toxoplasmosis. Epilepsy, another chronic condition, is often caused by a variety of infections that affect numerous low- and middle-income tropical countries—including toxoplasmosis. Earlier meta-analysis found only three studies and reported a 4.8-fold greater risk of epilepsy. This had many limitations, as discussed in this paper, our estimate is rather more robust, based on a higher number of studies, and corrects deficiencies that were present previously.

## Introduction

Epilepsy is a major chronic neurological disorder that affects about 70 million people worldwide [1]. However, its importance goes beyond mere numbers [2]. Most of its burden is felt in low- and middle-income tropical countries, where a number of infections that are important risk factors for epilepsy, are predominantly reported [3–5]. Parasitic infections are important causes of epileptic seizures or epilepsy, among other neurological and mental health conditions [6,7]. Infection with *Toxoplasma gondii (T. gondii*) in particular is reported to affect one-third of the world’s population, mainly in the low- and middle-income countries [8,9]. Certain currently available data strongly suggest the possibility of a relationship between toxoplasmosis and epilepsy [6] [10], although results to the contrary have also appeared [11]. Therefore, we conducted a systematic review and meta-analysis of published data to estimate the risk of epilepsy due to toxoplasmosis.

## Methods

### Literature search

To identify published studies on the association between toxoplasmosis and epilepsy, we conducted a systematic search of the literature published in English or French. The search was conducted on MEDLINE, INGENTACONNECT, REFDOC, SCIENCEDIRECT, GOOGLE, Médecine/Sciences, PLOS ONE and the database of the *Institut d’Epidémiologie neurologique et Neurologie Tropicale (IENT)*: http://www.unilim.fr/IENT/recherche_bvna.phpbase. This database contains medical dissertations, theses, and articles on tropical neurology and parasitology. The keywords for epidemiological aspects were: “*epilepsy*, *Toxoplasma*, *toxoplasmosis*”. Similar keywords were used in French. While searching, OR and/or AND were used for combination terms. We also searched the bibliographies of the articles retained after our database search.

### Selection of studies

Articles were selected based on their titles and then their abstracts. Those retained were read in full, but only those articles that met the inclusion-exclusion criteria were finally included. Inclusion criteria were: epilepsy as a disease and toxoplasmosis as an exposure, presence of a control group, sample sizes suitably estimated, details of techniques used to diagnose epilepsy and toxoplasmosis, and details on selection of participants, including socio-economic level. Data were then entered in a database covering: title, principal author, year of publication, type of study, objectives, methods, results, and any additional comments.

### Meta analysis

The meta-analysis was conducted using EasyMA version 2001 and Medcalc (Belgium) version 12.6.0. The measure of association between toxoplasmosis and epilepsy was a common odds-ratio (OR), recommended for the meta-analysis of observational studies. Respective ORs of each included study were individually verified. Random effects models were used to determine common odd ratios. Homogeneity of the studies was examined using Cochrane Q, 95% confidence intervals (CI) were also derived, and the statistical significance was set at 5%. A scatter plot was drawn from the combined data obtained.

## Results

**Fig 1:**
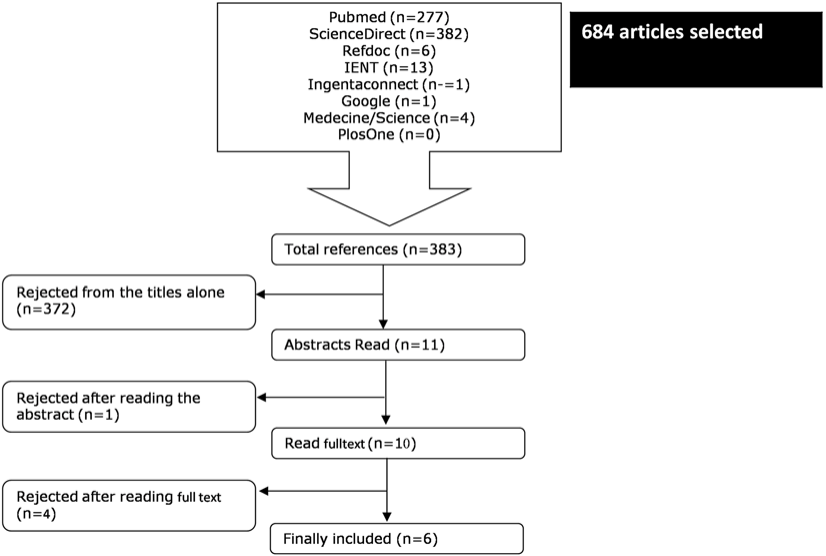
Flowchart of search of studies for epidemiological correlation between toxoplasmosis and epilepsy.

The results of the search are presented in Fig. 1. Five databases (Pubmed, ScienceDirect, Refdoc, IENT and Ingentaconnect), two journals (Médecine/Sciences, PlosOne) and Google search gave a total of 684 articles. Of these, 301 were cited more than once. Of the remaining 383 articles on toxoplasmosis and epilepsy, 372 articles were eliminated on the basis of title as either non-epidemiological or in a language other than English or French. The remaining 11 articles were read as abstracts, and one was excluded because its full text was not accessible. Furthermore, two were literature reviews [12] [13], one was a meta-analysis [10] and one was an epidemiological study that did not meet our inclusion criteria i.e. no control group [14]. The references of the review articles and meta-analysis did not add any new articles. Lastly, six studies were retained [2] [11] [3,15] [16] [17] [18], from Israel, USA, Turkey, Iran and various countries of sub-Saharan Africa. Interestingly, no studies were from Latin America, Europe, or most of Asia, table 1.

**Table 1:** Description of studies included in the meta-analysis of association between toxoplasmosis and epilepsy.

Reference	Study	Source 1	Definition	Source 2	Matching*	Measure of exposure
Potasman (Israel)	CC	Hospital	Other	Hospital	Unspecified	ELISA IgG (Tox-G EAI diagnostic kit, Abbott lab)
Stommel (USA)	CC	GP	ILAE	Community	Unspecified	ELISA IgG
Yazar (Turkey)	CC	Hospital	ILAE	Volunteers	Yes	ELISA IgG (ELISA Kit EUROIMMUN)
Akyol (Turkey)	CC	Hospital	ILAE	Volunteers	Yes	in house ELISA IgG kit
Zibaei (Iran)	CC	GP	ILAE	Community	Yes	Diaplus Inc, Toxo IgG, USA
Ngugi (SSA)	CC	GP	WHO	Community	Yes	IgG-ELISA, Genesis Diagnostics

CC: Case-control; GP: General population; ELISA: enzyme-linked immunosorbent assay; IgG: Immunoglobulin G; ILAE: International league against epilepsy; SSA: Sub-saharan Africa, WHO: World health organization,

*matching is by age and gender alone

Study: Study type; source 1: source of epilepsy cases; source 2: source of non-epilepsy controls; definition: definition of epilepsy; Other: clinical history by pediatric neurologist, repeated electroencephalography and computed tomography of the brain

As summarized in table 1, three studies recruited cases from hospital(s), but only one recruited controls from hospital(s). The remaining studies (n = 5) recruited controls from the community or used volunteers. Four studies matched controls for age and gender. Toxoplasma infection status was determined with the use of IgG ELISA, table 1. As summarized in table 2, only one study recruited subjects of all ages, as is often recommended. All but one study [11] reported a “risk relationship” between toxoplasmosis and the development of epilepsy, table 2. Only one of the included studies provided any urban-rural information [16].

**Table 2:** Description of data extracted from the included studies in the meta-analysis searching for an association between toxoplasmosis and epilepsy.

Reference	N	Age	EP+ (n)	EA- (n)	EP & T+(n, %)	EA & T+ (n, %)	OR (95% CI)	P-value
Potasman (1987–91, Israel)	161	1–15	52	109	10 (19.2%)	10 (9.0%)	2.36 (0.91–6.08)	0.11
Stommel (1997–99, USA)	45	adults	22	23	17 (75.0%)	13 (56.5%)	2.62 (0.72–9.54)	0.075
Yazar (1999–2002, Turkey)	100	adults	50	50	27 (54.0%)	9 (25.0%)	5.35 (2.15–13.30)	<0.001
Akyol (2003, Turkey)	150	11–64	100	50	31 (31.0%)	10 (20.0%)	1.80 (0.80–4.05)	0.2
Zibaei (2010, Iran)	170	7–62	85	85	12 (14.1%)	4 (4.7%)	3.33 (1.03–10.78)	0.036
Ngugi (2007–11, SSA)	2262	All	971	1291	380 (39.1%)	457 (35.4%)	1.17 (0.99–1.39)	0.06

Age is in years; CI: Confidence interval; EP+: People with epilepsy; EA-: People without epilepsy; EP & T+: People with epilepsy and *Toxoplasma* positive; EA & T+: People without epilepsy and *Toxoplasma* positive; OR: Odds ratio; SSA: Sub-saharan Africa

**Fig 2:**
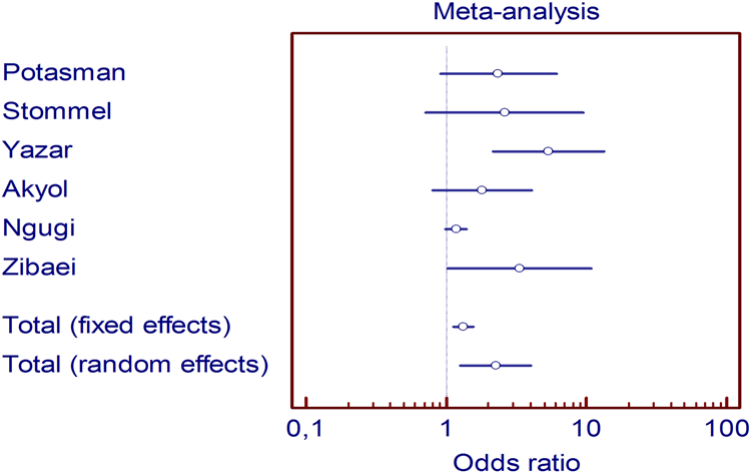
Scatter plot of odds ratios for epidemiological correlation between toxoplasmosis and epilepsy.

The total number of subjects in all included studies was 2888, of whom 1280 had epilepsy and 1608 did not (table 2). As summarized in table 2, the frequency of patients with epilepsy who were *Toxoplasm*a-positive varied from 14.1% to 75.0%. Among subjects without epilepsy, the proportion of *Toxoplasma* positivity varied between 4.70% and 56.50%. Seroprevalence for T. gondii was therefore higher among those who had epilepsy than those who did not. The ORs of included studies varied from 1.17 (95% CI 0.99–1.39) to 5.35 (95% CI 2.15–13.30) but only two of these results were significant (table 2). Scatter plots of the six studies are presented in Fig. 2. The common OR (fixed effects model) was 1.32 (95% CI 1.13–1.55), p<0.001. The heterogeneity was statistically significant, p = 0.006. Therefore, we estimated a common OR by a random effects model at 2.25 (95% CI 1.27–3.98), p = 0.05. The test of homogeneity was clearly not significant in this case, p = 0.6.

## Discussion

We conducted a systematic review and meta-analysis of literature primarily to estimate the relationship between chronic toxoplasmosis and epilepsy. Our literature search was comprehensive as different databases and specific journals were searched, including those in the French language to avoid any publication bias. However, this search might have excluded important studies published exclusively in other languages. The last meta-analysis on this subject, conducted in 2007, [10] included only three studies, whereas ours identified six, table 1.

One of the foremost lessons from our work is that despite the fact that *T.gondii* infection affects nearly one-third of the world’s population and is likely to be epileptogenic, few data exist on the subject. This certainly calls for more studies. Interestingly, none of the studies found were from Latin America, or Europe, and most of Asia remains unaddressed sparing two countries from Western Asia (Israel and Turkey) and one from Southern Asia (Iran), another important limitation. Toxoplasmosis in Asia is reportedly a silent threat [19], and epilepsy is far less well addressed in Asia than elsewhere. As seen in table 2, the OR was lower in African studies than those conducted elsewhere, whereas in Turkey OR was higher than elsewhere [11] [15]. Significant differences in the virulence and host response to strains are reported[20]. The extent to which these strain differences affect epileptogenic potential is not yet studied. Differences in strains can also be important in elucidating regional differences in the burden of toxoplasma-related epilepsy as there is a spatial distribution [20] [21] [22] [23] [24]. Strains circulating in Africa and South America are genetically quite different from those circulating in Europe or North America, and are responsible for more severe and frequent retinochoroiditis. It cannot be excluded that this higher pathogenicity observed in retinal tissue is also observed in brain tissue [20,24–26]. Very recently, a study conducted in Sub-Saharan Africa demonstrated that combinations of parasites can have additive effects on consequential conditions such as toxoplasmosis and onchocerciasis on active convulsive epilepsy [27].

Toxoplasmosis was found to be a risk factor for epilepsy; however the degree of risk differed between that reported in an earlier meta-analysis (OR 4.80, 95% CI 2.60–7.80) [10] and the one estimated by us (OR 2.25, 95% CI 1.15–3.93). The previous meta-analysis was, however based on only three studies [10].

Moreover, there were important differences in data extraction between the earlier meta-analysis [10] and ours. For instance, in one study [17], the number of patients with epilepsy was erroneously considered to be 95. The actual number was 52, as we calculated from the OR given in the study and the data recorded in the results [17]. A similar case relates to the estimated number of seropositives and seronegatives in another study [18]. The previous meta-analysis [10] estimated 18 seropositive cases and five seronegative controls, but this would give an OR of 12.24, higher than the 2.63 reported [18] However, we performed a calculation by approximation that permitted us to obtain, from the OR, the respective values of 10 and 13, which yielded an OR of 2.62, just about same as the original OR reported in that study.

Four studies out of six had non-significant results, table 2. Most had small sample sizes, with the exception of one by Ngugi et al [3]. This later study [3] yielded significant risk of epilepsy only in subjects >18 years of age, and not those <18 years old, table 2. Nevertheless, five out of six studies concluded that toxoplasmosis was a risk factor for epilepsy, while only one of our studies [11] failed to find any association between toxoplasmosis and epilepsy. However, this study had several limitations in its design and data analysis [28]. It is often contended that the burden of epilepsy moves proportionately with that of toxoplasmosis (e.g. more toxoplasmosis, more epilepsy) [10], but this may not be logically correct. Because of the large number of causes of epilepsy, one single factor like toxoplasmosis, about the epileptogenicity of which little is known, cannot explain such epidemiological shifts of a disease like epilepsy in any given population.

Also, there is always a possibility of an influence of variables such as 24 hour EEGs supervised by an epileptologist or diagnosis by simple history-taking by a family physician, etc., that may cause some influence on the diagnosis of epilepsy, hence on the results of some of these studies.

Various cellular, anatomical, immunological, and neurotransmitter-related changes occur as a result of infection with *T. gondii*, which plays a role in the development of various neurological conditions [29]. In general, the neuropathology of chronic latent infection differs from acute cerebral toxoplasmosis, in which necrosis and inflammation are generally widespread. For epilepsy, the neuropathology of *T. gondii* infection is yet to be fully elucidated and is speculative at best. Epileptogenic mechanisms of toxoplasmosis are probably multifactorial. Brain is one of the primary targets for the formation of *T. gondii* cysts and a variety of brain cells, particularly neurons, but microglia and astrocytes, can also get infected [30,31]. Neurons are predominantly infected because both astrocytes and microglia may protect themselves as a result of their ability to inhibit parasitic replication upon activation [32]. Once a chronic infection is established, *T. gondii* is found in tissue cysts containing the bradyzoite stage. Brain tissue cysts mature slowly; their wall may eventually rupture in immunocompetent hosts, liberating numerous bradyzoites that are capable of infecting new cells and inducing localized inflammation. This process may produce microglial scars (glial nodules). It has been suggested that scar tissue formation is one of the main causes of epilepsy in toxoplasmosis patients [10]. The likelihood of seizures would also depend on the location and numbers of cysts. Most cysts occur in the grey matter, a more epileptogenic area than white matter [33]. Other epilepsy-relevant areas such as the cerebral cortex, hippocampus, amygdala, and basal ganglia are also reported to be invaded by tissue cysts; while the greatest impact is reportedly on the hippocampus and amygdala [34]. In addition, the size of a cyst may also influence how it functionally impairs neuronal activity [35].

Neurotransmitters such as serotonin, glutamate, and gamma aminobutyric acid (GABA) need to be considered in *T. gondii*-induced neuropathological changes, and the host immune response to *T. gondii* infection can lead to altered neurotransmitter levels [36]. Chronic infection with *T. gondii* may also cause localized changes in serotonin levels [37]. The inhibitory neurotransmitter GABA is also important. In cases of infection with *T. gondii*, secretion of GABA increases as a response by dendritic cells to the invading parasite [38].

Data from animal studies show the following: intraneuronal *T. gondii* cysts directly modulate neuronal function, leading to either hypo- or hyper-responsive neurons [39,40] [41]. Tachyzoite infection of neurons results in dysregulation of calcium influx upon stimulation with glutamate [39], the major excitatory amino acid in the brain, which inevitably plays an important role in the initiation and spread of seizure activity. Another hypothesis concerns the importance of Ca^+^ ions in key aspects of the *T. gondii* life cycle, including motility, invasion and exit from the host cell [42]. Neuronal excitability can be influenced through depletion of calcium stores in the endoplasmic reticulum of the host cell. Uninfected neurons display a sustained calcium response, whereas infected neurons display either a short calcium response or complete failure. This indicates that *T. gondii* infected neurons exhibit depletion of calcium stores in the endoplasmic reticulum, and therefore influence the excitability of the neurons [43].

### Desirable criteria for good-quality studies on this subject

Good-quality epidemiological research will further help to draw broad conclusions on this important subject. These good-quality studies may at least be population-based with samples representative of all age-groups, and taking cases-controls from the same source population with at least 70% power. These studies should also use standard criteria to define various parameters and taking active epilepsy into account with clear description of procedures followed in the article.

### Conclusions

Few studies evaluating the risk of epilepsy following toxoplasmosis are available, and none cover Europe and Latin American regions. Most Asian countries also remain unaddressed except Israel, Turkey and Iran. Based on the currently available data, and their obvious limitations, it is still good to consider toxoplasmosis as a possible epilepsy risk factor, at least epidemiologically. However, many questions remain to be addressed in future studies. Combinations of parasites may have additive effects on consequential conditions such as toxoplasmosis and onchocerciasis on active epilepsy [27].

### Key learning points

- If one-third of the global human population carries a toxoplasma infection, and if a fraction of these toxoplasma carriers eventually develop epilepsy, then a huge number of people worldwide is at risk of developing toxoplasma-related epilepsy;
- Good quality studies are needed to correctly determine whether or not there is a risk/causal relationship between toxoplasmosis and epilepsy;
- Interestingly, there is no data from South America, Europe, or most of Asia, where more virulent *Toxoplasma* strains circulate;
- Based on current data, the risk of epilepsy with toxoplasmosis stands at 2.2-fold, but the confidence interval ranges between 1.27 and 3.9-fold;
- Epileptogenic mechanisms of toxoplasmosis are probably multifactorial (direct lodgment of parasite, direct modulation of neuronal functions, abnormalities in GABA, role of calcium, etc.);
- Many unanswered issues remain, including the role of different strains, defining suitable prevention strategies, defining epileptogenic mechanisms, etc.;
- Combinations of parasites may have additive effects on consequential conditions such as toxoplasmosis and onchocerciasis on active convulsive epilepsy.

### Key papers in the field

1. Palmer BS (2007) Meta-analysis of three case controlled studies and an ecological study into the link between cryptogenic epilepsy and chronic toxoplasmosis infection. Seizure 16: 657–663.
2. Stommel EW, Seguin, R., Thadani, V. M. et al. (2001) Cryptogenic epilepsy: an infectious aetiology?. Epilepsia 42: 436–438.
3. Veeranoot N (2007) Toxoplasmosis: A silent threat in Southeast Asia. Res J Parasitol 2: 1–12.
4. Fuks JM, Arrighi RB, Weidner JM, Kumar Mendu S, Jin Z, et al. (2012) GABAergic signaling is linked to a hypermigratory phenotype in dendritic cells infected by *Toxoplasma gondii*. PLoS Pathog 8: e1003051.
5. Haroon F, Händel U, Angenstein F, Goldschmidt J, Kreutzmann P, Lison H, Fischer KD, Scheich H, Wetzel W, Schlüter D, Budinger E. *Toxoplasma gondii* actively inhibits neuronal function in chronically infected mice. PLoS One. 2012;7(4):e35516.
6. Fischer HG, Nitzgen B, Reichmann G, Gross U, Hadding U (1997) Host cells of *Toxoplasma gondii* encystation in infected primary culture from mouse brain. Parasitol Res 83: 637–641.
7. Kamuyu G, Bottomley C, Mageto J, Lowe B, Wilkins PP, et al. (2014) Exposure to multiple parasites is associated with the prevalence of active convulsive epilepsy in sub-saharan Africa. PLoS Negl Trop Dis 8: e2908.

## Supporting Information

S1 Flow Diagram PRISMA 2009 Flow diagram. (DOC)

S1 Checklist PRISMA 2009 checklist. (DOC)
