# Supplementary material for: Toxoplasmosis and Epilepsy — Systematic Review and Meta Analysis
Source: PLoS Negl Trop Dis. 2015 Feb 19;9(2):e0003525. doi: 10.1371/journal.pntd.0003525 (PMC4335039; doi:10.1371/journal.pntd.0003525)
Supplement: S1 Flow Diagram — (DOC) [file pntd.0003525.s001.doc]

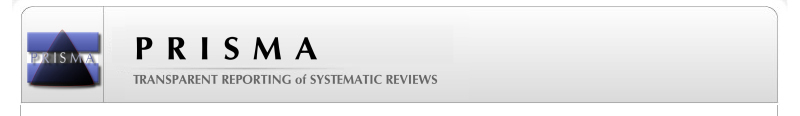
**PRISMA 2009 Flow Diagram**

**Screening**

**Included**

**Eligibility**

**Identification**

Records identified through database searching (Pubmed, Refdoc, IENT, Google)
(n =297)

Additional records identified through other sources (ScienceDirect, Ingentaconnect, Medecine/Science, PlosOne
(n = 387)

Records after duplicates removed
(n =0)

Records screened
(n = 383)

Records excluded from titles (n =373)

Full-text articles assessed for eligibility
(n =10)

Full-text articles excluded, with reasons
(n =4)

Studies included in qualitative synthesis
(n =6)

Studies included in quantitative synthesis (meta-analysis)
(n =6)
